# Bacterial Communities Associated with Atherosclerotic Plaques from Russian Individuals with Atherosclerosis

**DOI:** 10.1371/journal.pone.0164836

**Published:** 2016-10-13

**Authors:** Elvira E. Ziganshina, Dilyara M. Sharifullina, Andrey P. Lozhkin, Rustem N. Khayrullin, Igor M. Ignatyev, Ayrat M. Ziganshin

**Affiliations:** 1 Institute of Fundamental Medicine and Biology, Kazan (Volga Region) Federal University, Kazan 420008, The Republic of Tatarstan, Russia; 2 Interregional Clinical and Diagnostic Center, Kazan 420101, The Republic of Tatarstan, Russia; University of Illinois at Urbana-Champaign, UNITED STATES

## Abstract

Atherosclerosis is considered a chronic disease of the arterial wall and is the major cause of severe disease and death among individuals all over the world. Some recent studies have established the presence of bacteria in atherosclerotic plaque samples and suggested their possible contribution to the development of cardiovascular disease. The main objective of this preliminary pilot study was to better understand the bacterial diversity and abundance in human atherosclerotic plaques derived from common carotid arteries of individuals with atherosclerosis (Russian nationwide group) and contribute towards the further identification of a main group of atherosclerotic plaque bacteria by 454 pyrosequencing their 16S ribosomal RNA (16S rRNA) genes. The applied approach enabled the detection of bacterial DNA in all atherosclerotic plaques. We found that distinct members of the order *Burkholderiales* were present at high levels in all atherosclerotic plaques obtained from patients with atherosclerosis with the genus *Curvibacter* being predominant in all plaque samples. Moreover, unclassified *Burkholderiales* as well as members of the genera *Propionibacterium* and *Ralstonia* were typically the most significant taxa for all atherosclerotic plaques. Other genera such as *Burkholderia*, *Corynebacterium* and *Sediminibacterium* as well as unclassified *Comamonadaceae*, *Oxalobacteraceae*, *Rhodospirillaceae*, *Bradyrhizobiaceae* and *Burkholderiaceae* were always found but at low relative abundances of the total 16S rRNA gene population derived from all samples. Also, we found that some bacteria found in plaque samples correlated with some clinical parameters, including total cholesterol, alanine aminotransferase and fibrinogen levels. Finally, our study indicates that some bacterial agents at least partially may be involved in affecting the development of cardiovascular disease through different mechanisms.

## Introduction

Atherosclerosis is considered a chronic disease occurring at sites of blood flow disturbance and is the major cause of severe disease, loss of productive life years and death among individuals all over the world. This disease is characterized by accumulation of low-density lipoprotein in the arterial intima, expression of leukocyte adhesion molecules and chemokines by activated endothelium, promoting the recruitment of innate and adaptive immunity cells (monocytes and T cells) to the intima, thus resulting in local inflammation process. Further its intensification may lead to local proteolysis, atherosclerotic plaque disruption and ultimately to thrombus formation, leading to ischemia and myocardial infarction. Therefore, human atherosclerosis can be considered both a metabolic and an inflammatory disease [1–4].

The infectious hypothesis of atherosclerosis has been studied for some decades. Assuming involvement of both innate and adaptive immune systems in atherogenesis, infections with bacteria and/or viruses have been supposed to potentially contribute to the pathogenesis of atherosclerosis via direct and indirect mechanisms. Among infectious agents, which can present in plaques and have been proposed to have associations with cardiovascular disease, we can distinguish *Chlamydia pneumoniae*, *Helicobacter pylori*, some periodontopathogens, including *Porphyromonas gingivalis* and *Aggregatibacter actinomycetemcomitans*, as well as various viruses [5]. However, although the numerous researchers were able to find microbial infections and suggested them as a contributing factor in atherosclerosis pathogenesis, the strength of the evidence for most of the pathogens associated with cardiovascular disease is weak. Furthermore, antibiotic therapy of specific bacteria did not improve clinical outcomes of many individual patients with atherosclerosis [6], raising the question of implication of microorganisms in atherogenesis. However, Rosenfeld and Campbell [5] suggested that the failure of the antibiotic trials could be also due to persistent infection in the plaques and pathogen burden.

Application of modern molecular techniques allows a more comprehensive examination of taxonomically heterogeneous microbial communities [7–10]. Previous studies have identified genetic material of a broad diversity of bacteria in atherosclerotic plaques [11–19]. For example, Koren et al. [12] observed the predominance of *Chryseomonas*, *Staphylococcus*, *Propionibacterium* and *Burkholderia*, Wolcott et al. [13] found the high abundance of *Flavobacterium*, *Pseudomonas*, *Clostridium*, *Streptococcus* and *Acinetobacter*, Calandrini et al. [16] detected *Aggregatibacter actinomycetemcomitans* and *Pseudomonas* species as major phylotypes, whereas Mitra et al. [17] reported the dominance of *Lactobacillus rhamnosus*, *Neisseria polysaccharea*, *Helicobacter pylori* and *Waddlia chondrophila* in various atherosclerotic plaque samples. Such differences among various research works indicate that microbial associations in atherosclerotic plaques are highly diverse and variable between individual patients. Therefore, high-resolution investigations are extremely necessary to continue further identification of the main bacteria that may potentially play roles during the human atherosclerotic plaques development depending on previous and current diseases, lifestyle factors and obtained medical treatment. Moreover, it is still questionable whether these bacterial agents are at least partially involved in affecting the pathogenesis of atherosclerosis or they belong to only non-spreading microbial contaminants without the contribution to the inflammatory process of atherosclerosis.

Atherosclerosis usually remains silent as long as the arterial surface remains intact, whereas a breakdown of its integrity results in the formation of a thrombus resulting in clinical manifestation of this disease. Plaque rupture is caused by loss of mechanical stability. Mechanisms leading to endothelial erosion have been unclear, but recent research shows an involvement of innate immunity in this process [4]. Activation of Toll-like receptor-2 (TLR2) abundantly expressed by endothelial cells of atherosclerotic plaques contributes to endothelial apoptosis and denudation [20]. TLR2 ligands also include some components of gram-positive bacteria [21], indicating that some infectious factors may function to contribute to atherothrombosis via such mechanism [20]. These data indicate that infectious agents may be at least partially implicated in both pathogenesis of atherosclerosis and manifestation of cardiovascular disease proving that involvement of microorganisms in the processes described above requires additional studies.

The main goal of this pilot research was to better understand the bacterial diversity and its abundance in human atherosclerotic plaques derived from common carotid arteries of individual patients with atherosclerosis (Russian group) and contribute towards the further identification of a main group of atherosclerotic plaque bacteria (specific bacterial phylotypes) by pyrosequencing their 16S ribosomal RNA (16S rRNA) gene fragments.

## Materials and Methods

### Patients and inclusion criteria

Blood and tissue samples were obtained from twenty eight patients aged 54–76 years (Russian nationwide group), who underwent the carotid endarterectomy in the Vascular Surgery Department of Interregional Clinical and Diagnostic Center (Kazan, Russia). Twenty eight patients had previously received a diagnosis of atherosclerosis in the common carotid arteries. The main characteristics of study participants are presented in Table 1. Blood samples were collected and analyzed before surgery as described below. The segments of unstable atherosclerotic plaques were removed during surgery and placed directly into sterile containers under strict aseptic conditions. After surgery, all obtained plaque samples did not get in contact with any potential contaminants. The plaque samples were then immediately transferred into a –70°C freezer in the Vascular Surgery Department. The patients completed their questionnaires regarding previous and current diseases, lifestyle factors and medical treatment.

**Table 1:** Some characteristics of study individuals.

Data	Value
Patients, n	28
Age, years, mean ± SD	64.3 ± 6.51
Male/female	19/9
Body mass index, mean ± SD	27.98 ± 3.79
Current smoker, n (%)	7 (25.0)
Diabetic, n (%)	11 (39.3)
Hypertension, n (%)	25 (89.3)
Myocardial infarction, n (%)	4 (14.3)
Stroke, n (%)	12 (42.9)
Statin treatment, n (%)	17 (60.7)
Antiplatelet therapy, n (%)	19 (67.9)

### Ethics statements

This study was approved by Ethics Committee of Interregional Clinical and Diagnostic Center (Kazan, the Republic of Tatarstan, Russia), and all studied patients signed a consent form prior the study begin.

### Blood drawing and sample processing

Blood samples were collected before surgery under aseptic conditions without venous stasis using blood collection vacuum tubes, stored at room temperature and analyzed within 2 hours. The first blood samples were added to 3.2% trisodium citrate (9:1 by volume) in S-Monovette tubes (Sarstedt, Germany) and then centrifuged at 1,500×*g* for 10 min to obtain platelet-poor plasma for further blood coagulation tests. The second blood samples were stabilized with 1.6 mg EDTA/mL blood (final concentration) tripotassium salt of ethylenediaminetetraacetic acid (K3-EDTA) and used for hematological tests. A non-stabilized whole blood samples were thoroughly mixed with beads coated with a clotting activator (Sarstedt, Germany), left for 20–30 minutes at 37°C to allow clotting followed by centrifugation step (2,000×*g*, 10 min) to obtain serum that was further used for biochemical blood tests.

### Coagulation, hematological and blood chemistry tests

The Sysmex CA-1500 system (Japan) and fresh citrated plasma samples were used for the following tests: activated partial thromboplastin time (APTT), international normalized ratio (INR), thrombin time (TT) and concentration of fibrinogen. Cell count was performed in EDTA whole blood samples with a hematology analyzer ABX Pentra 60 (Horiba, Japan). The following parameters were analyzed: hemoglobin, erythrocyte, platelet, total leukocyte, neutrophil, eosinophil, basophile, lymphocyte and monocyte counts. Blood chemistry analyses were performed using an RX Imola (Randox, UK) and an Advia 1200 (Siemens, Germany) chemistry analyzers (Siemens, Germany). Glucose concentration, alanine aminotransferase (ALT), aspartate aminotransferase (AST), creatinine and urea were measured with the RX Imola analyzer. Cholesterol concentration analysis was performed on the Advia 1200 analyzer.

### DNA extraction and 16S rRNA gene pyrosequencing

Total genomic DNA from the human atherosclerotic plaque samples was extracted by using a Fast DNA SPIN Kit (MP Biomedicals, USA). All samples were homogenized with a SuperFastPrep-1 (MP Biomedicals, USA) at maximum speed 2 times for 40 sec. The integrity of the recovered genomic DNA was evaluated by agarose gel electrophoresis, and the DNA concentrations were determined with the Qubit dsDNA BR assay kit and a Qubit 2.0 Fluorometer (Invitrogen, Carlsbad, CA).

The V1–V3 regions of the 16S rRNA gene were PCR amplified with the barcoded primers 27F (5′-GAGTTTGATCCTGGCTCAG-3′) and 533R (5′-TTACCGCGGCTGCTGGCAC-3′) and pyrosequenced using the GS Junior System (454 Life Sciences, Roche) as described previously [22–24]. Briefly, PCRs were conducted in triplicate (using the same MID for each sample, then pooled) in 30 μl reactions with the FastStart High Fidelity PCR System (Roche, Branford, CT). The PCR products were then cleaned using the Agencourt AMPure XP system (Beckman Coulter, Brea, CA, USA) and analyzed on a 2100 Bioanalyzer (Agilent Technologies, Santa Clara, CA, USA) following the manufacturers’ instructions. Amplicon sequencing of bacterial 16S rRNA gene fragments was carried out following the manufacturer’s recommendations at the Interdisciplinary Center for Collective Use of Kazan Federal University (Kazan, Russia).

### Bioinformatic processing, data and statistical analyses

The 16S data were further analyzed using the bioinformatics pipeline QIIME, version 1.9.1 [25]. FlowClus was used as a systematic approach to filter and denoise the received reads [26]. Sequences that were shorter than 150 bp were removed from the pyrosequencing-derived datasets, and read end sections of 50 bp dropping below the quality score threshold of 20 were trimmed. Probable chimeric sequences were identified using an implemented USEARCH 6.1 [27] and further removed. Sequences were clustered into operational taxonomic units (OTU) (at least five reads for an OTU) based on the 97% identity threshold (reference-based OTU picking followed by de novo OTU clustering of sequences). For the taxonomic classification, the latest Greengenes reference (release v.13_8) [28] and RDP Classifier 2.2 were used. Observed OTU numbers, Simpson, Chao 1 and Shannon diversity indices along with the phylogenetic diversity were calculated as the indicators for alpha diversity. To determine beta diversity among all atherosclerotic plaque samples, beta diversity matrices were performed using UniFrac metrics implemented in QIIME with following principal coordinate analysis (PCoA). Heat map of the relative abundances of OTUs in individual atherosclerotic plaques was created with R package. Correlations between the abundances of different taxa in atherosclerotic plaque samples and various clinical values were analyzed using R Hmisc package (based on the Spearman’s rank correlation coefficient). The obtained pyrotag sequences were deposited under the EMBL-EBI accession number PRJEB12836 (http://www.ebi.ac.uk/ena/data/view/PRJEB12836). In addition, GraphPad Prism 5 as well as Microsoft Excel 2013 were used for data analyses and their interpretation.

## Results

### Atherosclerotic plaques were associated with high microbial diversity

**Fig 1:**
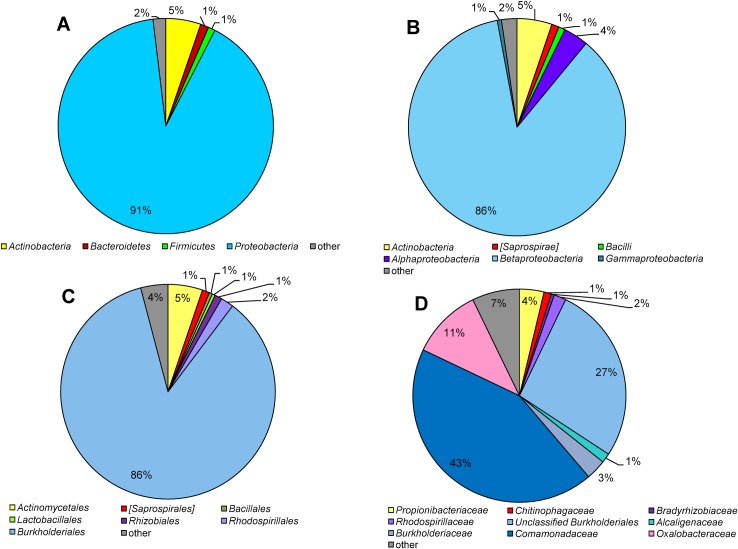
Bacteria associated with atherosclerotic plaque samples (mean values). Taxonomic classification of bacterial reads at (A) phylum level, (B) class level, (C) order level and (D) family level is demonstrated (data show more than 0.5%). Representatives accounting for less than 0.5% of all classified sequences are summarized in the group *“others”*.

The bacterial community composition in atherosclerotic plaques derived from common carotid arteries of twenty eight individual patients with clinical atherosclerosis was analyzed by V1–V3 16S rRNA gene region pyrosequencing analysis. The main characteristics of study participants are presented in Table 1 and S1 Table. A total of 249,876 bacterial sequences without chimeras were generated after processing from twenty eight samples. The read numbers varied, ranging from 5,552 to 13,841 per sample (Table 2). Since species richness increases with the number of obtained sequences in a sample, we subsampled all twenty eight atherosclerotic plaque samples to the similar size based on the sample having the smallest sequences number (~5,500 reads) for direct comparison. Finally, out of 154,428 reads, 146,407 reads were assigned to 1017 OTUs (139–319 OTUs per sample). The final data group included members of 17 bacterial phyla. The most of the resulted sequences were classified as *Proteobacteria* (90.5%), *Actinobacteria* (5.3%), *Bacteroidetes* (1.2%) and *Firmicutes* (1.1%). Fig 1 demonstrates the taxonomic classification of all received bacterial reads at phylum, class, order and family taxa levels (these data show more than 0.5%).

**Table 2:** Bacterial community α diversity within atherosclerotic plaque samples.

Sample	Number of reads without chimeras	Number of rarefied reads	Observed OTUs	Phylogenetic diversity	Chao 1 index	Shannon index	Simpson index
ap01	6,376	5,566	298	8.66	413.94	5.23	0.92
ap02	6,890	5,505	269	9.86	335.07	4.73	0.88
ap03	6,289	5,527	265	11.05	299.92	4.88	0.87
ap04	6,325	5,474	239	9.02	310.50	5.21	0.93
ap05	8,166	5,448	296	8.44	350.01	4.90	0.87
ap06	11,479	5,492	319	7.60	444.67	4.45	0.83
ap07	13,841	5,513	308	8.24	382.17	5.73	0.95
ap08	8,534	5,483	253	8.68	306.63	4.54	0.85
ap09	5,552	5,552	258	8.98	317.38	4.41	0.84
ap10	5,637	5,516	283	8.25	362.33	5.13	0.92
ap11	7,184	5,547	171	7.85	256.55	3.14	0.73
ap12	8,608	5,565	139	7.18	194.65	3.00	0.73
ap13	6,856	5,452	175	11.22	222.53	3.90	0.80
ap14	7,912	5,612	183	8.48	234.11	3.48	0.78
ap15	10,781	5,530	162	9.60	231.79	3.09	0.72
ap16	9,878	5,429	165	9.65	206.13	3.16	0.73
ap17	7,833	5,528	178	10.07	252.25	3.42	0.76
ap18	8,337	5,457	176	8.81	227.85	3.16	0.73
ap19	9,964	5,468	201	9.72	250.11	3.78	0.82
ap20	7,332	5,487	169	9.93	222.01	3.48	0.78
ap21	8,636	5,541	173	8.10	207.03	3.48	0.77
ap22	10,903	5,451	180	7.09	247.20	3.49	0.77
ap23	10,657	5,513	173	7.89	224.04	3.84	0.81
ap24	10,808	5,559	182	9.01	225.97	3.66	0.79
ap25	12,777	5,510	156	7.92	215.63	3.24	0.75
ap26	13,030	5,535	177	8.77	253.96	3.59	0.78
ap27	8,215	5,541	159	9.15	198.60	3.39	0.76
ap28	11,076	5,627	168	9.90	194.56	3.74	0.81

**Fig 2:**
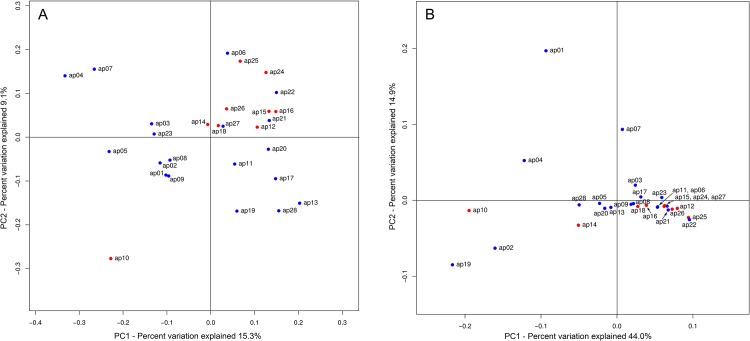
PCoA plots of the bacterial communities distribution in atherosclerotic plaque samples based on the data received by pyrosequencing of 16S rRNA gene fragments. (A) Unweighted Unifrac measurements. (B) Weighted Unifrac measurements (blue circles–men patients, red circles–women patients).

Bacterial diversity within each sample (alpha diversity) was calculated at given numbers of reads (n = ~5500), including number of observed OTUs, phylogenetic diversity, Simpson index, Chao 1 index and Shannon index (Table 2). In general, the obtained data indicate that bacterial communities of all samples were diverse and rich. The variation in overall bacterial community composition based on unweighted and weighted UniFrac distance measurements (beta diversity) was compared within all patients’ samples (Fig 2). In accordance with principal coordinate analysis (unweighted and weighted UniFrac distance metrics), almost all analyzed samples derived from women patients were closely spotted on the PCoA plots with grouping with each other. In turn, some samples derived from men patients were relatively close to each other, whereas other samples were separately spotted on the PCoA plots without grouping with each other (Fig 2). The received results indicate that bacterial communities among some atherosclerotic plaque samples did not vary significantly while other plaque samples had diverse bacterial communities.

### Characterization of bacteria in atherosclerotic plaques

**Fig 3:**
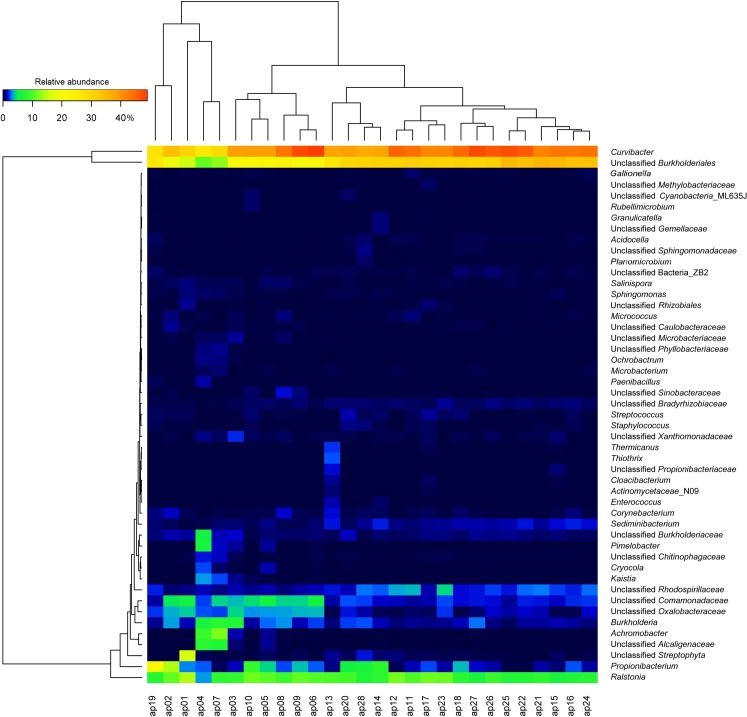
Heat map of the relative abundances of OTUs which differed between individual atherosclerotic plaque samples.

The applied sequencing approach enabled the detection of bacterial communities in atherosclerotic plaques (Fig 1). The bacterial associations were most commonly dominated by members of the phylum *Proteobacteria* (71.4–97.3%) (mostly by *β*-proteobacteria). Further phyla present in all plaque samples but in different proportions were *Actinobacteria* (0.2–24.1%), *Bacteroidetes* (0.3–2.1%) and *Firmicutes* (0.1–5.2%). Other phyla such as *Acidobacteria*, *Chloroflexi*, *Cyanobacteria*, *Gemmatimonadetes*, *Planctomycetes*, *Spirochaetes*, *Tenericutes*, *Verrucomicrobia* and candidate divisions GN02, OD1, SBR1093, TM6 and TM7 were found in some samples but in very minor proportions. The orders *Burkholderiales* (67.0–94.1%), *Actinomycetales* (0.2–23.2%), *[Saprospirales]* (0.3–2.1%) and *Bacillales* (0.1–3.6%) were the important taxa within the phyla *Proteobacteria*, *Actinobacteria*, *Bacteroidetes* and *Firmicutes*, respectively. Fig 3 demonstrates a heat map of the relative abundances of OTUs which differed within individual atherosclerotic plaque samples (genus/higher taxa level). *Curvibacter* (26.5–47.8%), *Propionibacterium* (0.1–21.8%), *Ralstonia* (3.2–14.0%), *Burkholderia* (0.4–7.9%) as well as unclassified *Burkholderiales* (11.0–35.9%), unclassified *Comamonadaceae* (1.0–5.8%) and unclassified *Oxalobacteraceae* (0.2–4.2%) were typically the most significant taxa for all atherosclerotic plaques, whereas different other taxa were also observed (Figs 1 and 3). Other genera, such as *Corynebacterium* and *Sediminibacterium* as well as unknown and unclassified *Rhodospirillaceae*, *Bradyrhizobiaceae* and *Burkholderiaceae* were always found but at low relative abundances of the total 16S rRNA gene population determined from all samples. In addition, *Achromobacter*, *Pimelobacter*, unclassified *Streptophyta* and unclassified *Alcaligenaceae* were detected in significant proportions but only in some samples (Fig 3).

### Correlation of clinical values with bacterial community structure

The relationships between bacterial community members and various blood parameters were investigated by correlation analysis. The applied analysis revealed that abundances of several OTUs correlated with different test parameters. The major outcome is described below while the complete correlation data set is demonstrated in S1 Fig. Thus, the abundances of *Microbacterium*, *Achromobacter*, *Pimelobacter*, unclassified *Microbacteriaceae* and *Alcaligenaceae* were negatively correlated with the total cholesterol concentration (ρ = –0.58, –0.58, –0.52, –0.52 and –0.50, respectively), whereas opposed relationships were found for *Granulicatella* (ρ = 0.57). Alanine aminotransferase value (ALT) was found to be significant factor for *Staphylococcus*, *Streptococcus* and *Propionibacterium* (ρ = 0.71, 0.53 and 0.50, accordingly). Unclassified *Rhodospirillaceae* showed a positive correlation with the urea level (ρ = 0.51), whereas *Burkholderia* demonstrated a negative correlation with this parameter level (ρ = –0.51). Unclassified *Burkholderiales* positively correlated with the fibrinogen level (ρ = 0.52), whereas unclassified *Burkholderiaceae* negatively correlated with the level of fibrinogen (ρ = –0.67). Moreover, abundance of unclassified *Methylobacteriaceae* was negatively correlated with the platelets level (ρ = –0.58), abundance of unclassified *Microbacteriaceae* was positively correlated with the neutrophils counts (ρ = 0.51), and additionally abundance of *Streptococcus* was negatively correlated with the level of lymphocytes and monocytes (–0.52 and –0.52, respectively) (S1 Fig).

## Discussion

In the present preliminary pilot study we used a 16S rRNA gene amplicon pyrosequencing approach to comprehensively describe the structure and diversity of the bacterial communities in atherosclerotic plaques derived from common carotid arteries of Russian individuals with atherosclerosis in greater detail. Correlations between bacterial community composition and some clinical values were additionally investigated. The research described herein clearly shows that microbial associations are present in atherosclerotic plaques and, on the one hand, represented by certain bacterial phylotypes in all plaques and, on the other hand, represented by diverse phylotypes across individual patients.

Despite the detection of bacteria in plaques in some other studies [11–17], our findings demonstrate the prevalence of some other distinct bacterial groups in atherosclerotic plaques. For example, Koren et al. [12] observed the predominance of *Chryseomonas*, Wolcott et al. [13] found the high abundance of *Flavobacterium* sp., Calandrini et al. [16] detected *Aggregatibacter actinomycetemcomitans* as major phylotypes, whereas Mitra et al. [17] reported the dominance of *Lactobacillus rhamnosus* in various atherosclerotic plaque samples. In the present research work all these bacteria were detected at very low levels or could not be detected at all. Such differences observed within various research works indicate that microbial associations in atherosclerotic plaques are highly diverse and variable between individuals. Therefore, such experiments are extremely necessary to identify key microorganisms that may potentially play roles during the atherosclerotic plaques development in humans depending on previous and current diseases, lifestyle factors and obtained medical treatment. In addition, some researchers analyzed other regions of the bacterial 16S rRNA gene (e.g., V1–V2 [12], V3–V5 [15], the full-length 16S rRNA gene [14]) as well as used other data analysis techniques, which could have additionally led to the differences observed in bacterial community content of various atherosclerotic plaques. Furthermore, Mitra et al. [17] used whole-genome shotgun sequencing approach, which might have helped them to obtain greater picture of the bacterial community composition.

We found that distinct members of the order *Burkholderiales* were present at high levels in all atherosclerotic plaques studied here with the genus *Curvibacter* being predominant in all analyzed samples. Bacteria belonging to the order *Burkholderiales* can be found in various environments including soil and water. Several species of this order have resistance to common antibiotics and are also perilous for different patients, e.g., with chronic lung diseases [29]. At the present time, the bacterial genus *Curvibacter* includes such recognized species as *Curvibacter delicatus*, *Curvibacter gracilis*, *Curvibacter lanceolatus* [30] and *Curvibacter fontanus* [31]. They are microaerobic mesophilic bacteria previously isolated from various aqueous environments such as well water. The presence of the genus *Curvibacter* in low proportions (below 5%) in some atherosclerotic plaque samples was also reported by Wolcott et al. [13]. Furthermore, the prevalence of members of the genus *Curvibacter* was observed in patients with chronic obstructive pulmonary disease compared to healthy individuals [32].

Besides the dominant genus *Curvibacter*, we also detected *Ralstonia* and *Burkholderia* within the order *Burkholderiales* in all analyzed samples. Species of the aerobic genus *Ralstonia* can be found in distinct ecological niches, e.g., *Ralstonia solanacearum* is a soil-borne pathogen causing diseases of many plant species and belonging to one of the most devastating bacterial plant pathogens [33]. *Ralstonia eutropha* can switch to anaerobic respiration under anoxic conditions, exploiting alternative electron acceptors [34]. Another representative of the same genus, *Ralstonia pickettii*, has been found in distinct clinical environments and recognized as potential nosocomial agent [35]. In addition, these bacteria have been recovered from various water sources, including municipal drinking water supplies [36] and bottled mineral water [37]. *R*. *pickettii* can cause minor infections as well as invasive infections such as sepsis or meningitis [38]. *R*. *pickettii*, *Ralstonia insidiosa* as well as *Ralstonia mannitolilytica* have also been recovered from the respiratory tract of cystic fibrosis patients [39, 40]. Falcone-Dias et al. [37] suggested that bacteria with the highest multi-resistance indices, including several representatives of the genera *Ralstonia* and *Curvibacter*, could be potentially transmitted to humans from bottled mineral water. *Ralstonia* genus was also observed in some atherosclerotic plaques in patients with and without chronic periodontitis using cloning and sequencing of 16S rRNA gene fragments [14, 15].

Our molecular analysis additionally revealed the presence of *Burkholderia* phylotypes in all plaque samples. *Burkholderia cepacia* is resistant to many antibiotics and disinfectants and is mostly associated with infections in patients having lung diseases (e.g., with cystic fibrosis and chronic granulomatous disease) as well as in hospitalized patients and individuals having impaired immune systems [41]. In addition, one OTU classified as microaerophilic, anaerobic *Propionibacterium acnes* (the order *Actinomycetales*) was specific for atherosclerotic plaques and present in all samples. *P*. *acnes* has been described as a member of the normal microflora of the skin, oral cavity and conjunctiva in humans [42]. However, this organism has also been associated with various infections, including endocarditis, intravascular infections [43, 44] as well as endophthalmitis [45]. Interestingly, that *Burkholderia* spp. as well as *P*. *acnes* were specific for various atherosclerotic plaques investigated in previous works [12, 13, 15]. Also, previously we were able to isolate and cultivate some *P*. *acnes* strains from atherosclerotic plaques of patients with atherosclerosis [46]. Except for phylotypes assigned to the genus level, we were able to detect various unclassified *Burkholderiales*, unclassified *Comamonadaceae* and unclassified *Oxalobacteraceae* at significant levels, which limited our ability to describe their potential roles in organisms. *Achromobacter* species (the order *Burkholderiales*) found at some levels in several our atherosclerotic plaque samples have also been recovered previously, e.g., from lungs of patients with cystic fibrosis [47] and patients with renal abscess [48].

Moreover, we found that several groups of microorganisms found in various proportions in atherosclerotic plaque samples correlated with some coagulation, hematological and blood chemistry test analyses. For example, the abundances of the genera *Microbacterium*, *Achromobacter*, *Pimelobacter* as well as unclassified *Microbacteriaceae* and *Alcaligenaceae* were negatively correlated with the plasma cholesterol level, whereas the abundance of *Granulicatella* was positively correlated to the level of this disease marker. *Granulicatella* spp. belong to normal components of the oral microflora, but have also been associated with different invasive human infections and noted as a cause of bacterial endocarditis [49]. Furthermore, ALT value was found to be significant factor for the genera *Staphylococcus*, *Streptococcus* and *Propionibacterium*. All these members are part of the normal flora in humans but may be also considered as major pathogenic bacteria of humans. For example, *Streptococcus* spp. and *Staphylococcus* spp. can cause endocarditis, bacteremia, pneumonia, meningitis as well as other infectious diseases [50, 51]. *Staphylococcus* isolates were also recovered and cultivated from some atherosclerotic plaques of patients with atherosclerosis what was shown in our recent study [46]. However, the abundances of some major phylotypes were not significantly correlated with most of the applied blood parameters.

Despite the fact that bacteria were identified in several atherosclerotic plaques in numerous studies [11–17, 52], there is still no clear understanding of whether these bacterial agents are at least partially involved in affecting the pathogenesis of atherosclerosis or these bacteria belong to only non-spreading microbial contaminants without the contribution to the inflammatory process of atherosclerosis. Since the treatment with antibiotics of many patients with atherosclerosis did not lead to improvement of clinical outcomes [6], this may have resulted in erroneous skepticism about bacteria being involved in the pathogenesis of atherosclerosis. Furthermore, Falcone-Dias et al. [37] suggested that bacteria having resistance to four or more classes of antibiotics could be potentially transmitted to humans from bottled water. In this research we identified different significant bacterial phylotypes in all analyzed atherosclerotic plaque samples. Therefore, the abundance of such bacteria in atherosclerotic plaques analyzed in our study might be also due to their higher rates of antibiotic resistance and the quality of water supply systems. However, we cannot fully exclude a risk of partial microbial contamination of some plaque samples during surgery. Also, the presence of several bacteria on atherosclerotic plaques might be the result of the bacterial colonization of injured vascular walls (such as in endocarditis).

In most cases bacteria encased in a biofilm matrix become more resistant to distinct antibiotics and the host immunity system (e.g., antibodies, leukocytes and complement) than are planktonic forms of the same bacteria, potentially increasing the current problems related to medical biofilms [53], and particularly to bacterial biofilms possibly associated with the pathogenesis of atherosclerosis [13]. After adhesion to a surface, bacteria can secrete mainly insoluble exopolymers, forming a biofilm matrix. From a medical point of view, both commensal and pathogenic microorganisms are able to form biofilm conglomerates that can be attached to various surfaces [53]. Therefore, in addition to the established factors of pathogenesis of atherosclerosis (accumulation of cholesterol and attack of macrophages on the arterial wall [54]), such bacterial biofilm structures may additionally contribute to the persistent inflammation associated with the pathogenesis of atherosclerosis, but do not prove that bacterial communities are the direct cause of atherosclerosis. Bacteria (single cells or fragments of biofilms) are able to enter the vascular system via different mechanisms under normal and inflamed conditions [55]. All these bacteria are then quickly cleared by the immune system of organism but some biofilm fragments with colony defenses mechanisms may attach to damaged endothelial cells [13]. Different bacterial agents could secondarily colonize atheromatous lesions and act as an additional factor accelerating coronary heart disease progression [56].

Furthermore, Koren et al. [12] suggested that infected macrophages at epithelial linings (e.g., oral cavity, gut and lung) may specifically target various bacteria to atheromas. Also, Mitra et al. [17] suggested that bacterial DNA found in atheromas could represent DNA from bacterial cells engulfed and killed in the body by phagocytic cells. Macrophages belong to one of the most abundant cell types in plaques and play important role in the development of atherosclerosis (one type of arterial surface damage) [54]. Koren et al. [12] demonstrated that the oral cavity and gut can be sources for bacteria associated with human atherosclerotic plaques. Moreover, there is a rising evidence of a relationship between periodontal microbiota and atherosclerosis in humans [15, 16, 57, 58] and mice [59]. Hansen et al. [57] showed that patients who received a hospital diagnosis of periodontitis had a high level of co-morbidity and an increased risk of cardiovascular disease and all-cause mortality. However, periodontopathogens were rarely identified in our samples.

Mechanisms resulting in endothelial erosion (another type of arterial surface damage) have been unclear; however, recent research works demonstrate an involvement of innate immunity in this process. Ligation of TLR2 expressed by endothelial cells overlying atherosclerotic plaques contributes to endothelial apoptosis and denudation [20]. TLR2 ligands include some components of bacterial pathogens as well as hyaluronan released from the extracellular matrix [21], indicating that infectious and endogenous factors may function to contribute to atherothrombosis via such mechanism [20]. These data indicate that some infectious agents found in our research may be at least partially implicated in the development of cardiovascular disease.

The human organism is home to various microbial ecosystems which perform distinct functional roles. In the present research, utilizing a 16S rRNA gene amplicon pyrosequencing approach, we revealed that various representatives of the order *Burkholderiales* were present at significant levels in all atherosclerotic plaques obtained from Russian patients with atherosclerosis. They as well as several other bacteria from 17 bacterial phyla may represent main members of atherosclerotic plaque consortia. Also, we found that several microbes found in atherosclerotic plaque samples correlated with some clinical parameters, including total cholesterol, ALT and fibrinogen levels. Future investigations such as isolation of specific microorganisms from atherosclerotic plaques, their further accurate investigation (e.g., origin, resistance to various antimicrobial agents), metatranscriptome analysis as well as microbial characterization of atherosclerotic plaques of other localization in humans should clarify whether these microorganisms at least partially affect the pathogenesis of atherosclerosis.

## Supporting Information

S1 Fig Correlation analysis of the atherosclerotic plaque bacterial community with various blood test parameters of study participants. Correlation analysis is based on the Spearman’s rank correlation coefficients which are shown by color ranging. Negative correlations are displayed in red color while positive correlations are displayed in green color. Significant correlations are marked by *P < 0.01 and **P < 0.001. Abbreviations: alanine aminotransferase (ALT), aspartate aminotransferase (AST), activated partial thromboplastin time (APTT), thrombin time (TT) and international normalized ratio (INR).
(PDF)

S1 Table Characteristics of study participants. (PDF)
