# Supplementary figures and images for: Bacterial Communities Associated with Atherosclerotic Plaques from Russian Individuals with Atherosclerosis
Source: PLoS One. 2016 Oct 13;11(10):e0164836. doi: 10.1371/journal.pone.0164836 (PMC5063344; doi:10.1371/journal.pone.0164836)

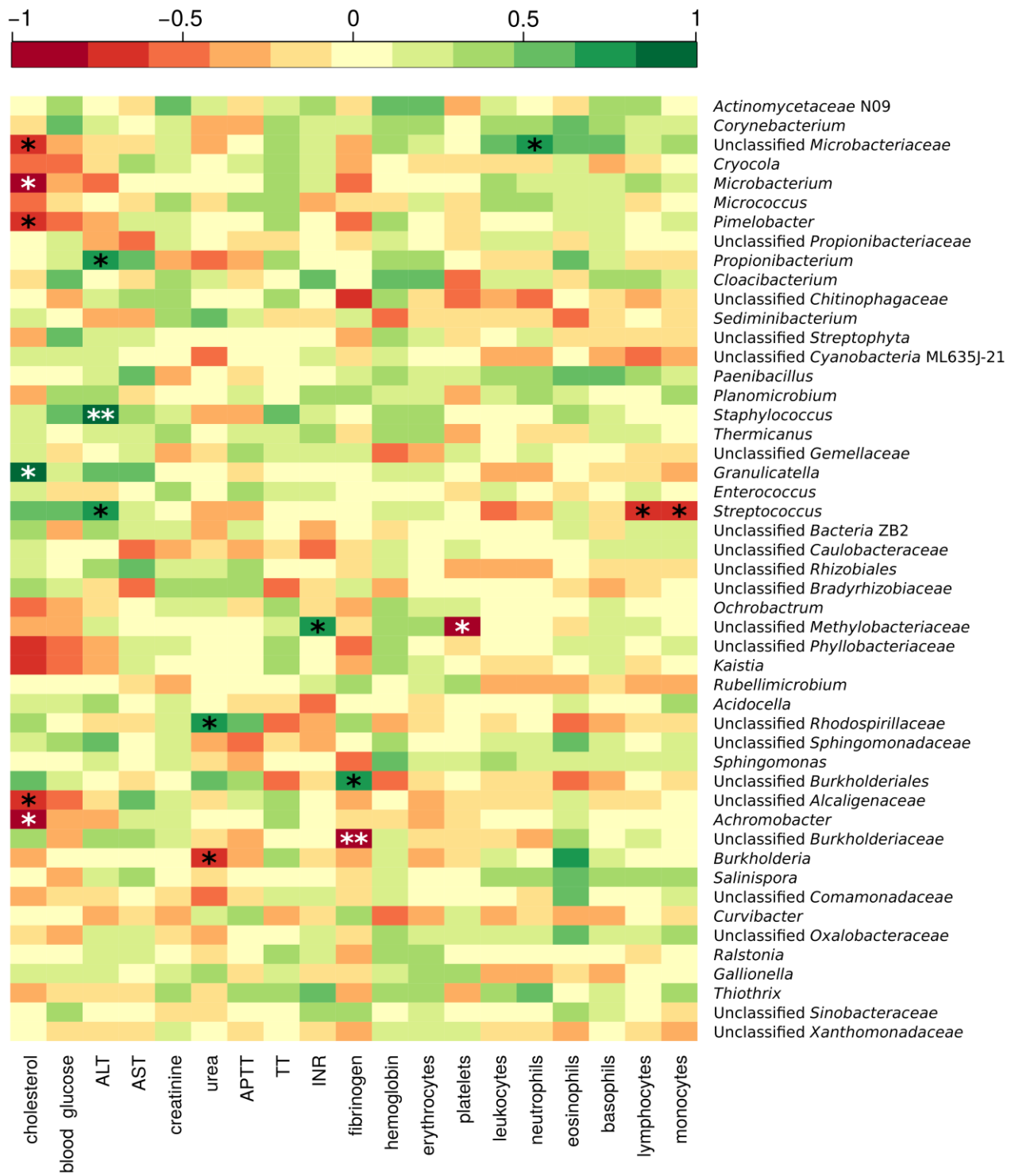

**Fig S1.**

Supplement: S1 Fig — Correlation analysis is based on the Spearman’s rank correlation coefficients which are shown by color ranging. Negative correlations are displayed in red color while positive correlations are displayed in green color. Significant correlations are marked by *P < 0.01 and **P < 0.001. Abbreviations: alanine aminotransferase (ALT), aspartate aminotransferase (AST), activated partial thromboplastin time (APTT), thrombin time (TT) and international normalized ratio (INR). (PDF) [file pone.0164836.s001.pdf]
