# Supplementary material for: Bacterial Communities Associated with Atherosclerotic Plaques from Russian Individuals with Atherosclerosis
Source: PLoS One. 2016 Oct 13;11(10):e0164836. doi: 10.1371/journal.pone.0164836 (PMC5063344; doi:10.1371/journal.pone.0164836)
Supplement: S1 Table — (PDF) [file pone.0164836.s002.pdf]

Table S1.

	Values	Patients (n = 28)
Blood chemistry tests	Total cholesterol, mmol/L	5.05 ± 1.39
	Blood glucose, mmol/L	5.73 ± 1.46
	Alanine aminotransferase (ALT), u/L	23.90 ± 13.09
	Aspartate aminotransferase (AST), u/L	23.59 ± 7.27
	Creatinine, μmol/L	90.13 ± 18.79
	Urea, mmol/L	6.05 ± 1.48
Coagulation tests	Activated partial thromboplastin time (APTT), sec	31.69 ± 2.98
	Thrombin time (TT), sec	18.95 ± 1.38
	International normalized ratio (INR)	0.99 ± 0.06
	Fibrinogen, g/L	3.54 ± 0.95
Hematological tests	Hemoglobin, g/L	145.00 ± 13.36
	Erythrocytes, $\times 10^{12}$/L	4.61 ± 0.36
	Platelets, $\times 10^{9}$/L	293.73 ± 94.67
	Leukocytes, $\times 10^{9}$/L	7.32 ± 2.33
	Neutrophils, $\times 10^{9}$/L	4.14 ± 1.42
	Eosinophils, $\times 10^{9}$/L	0.24 ± 0.19
	Basophils, $\times 10^{9}$/L	0.05 ± 0.03
	Lymphocytes, $\times 10^{9}$/L	2.30 ± 0.92
	Monocytes, $\times 10^{9}$/L	0.59 ± 0.21
